# Progress to Eliminate Trachoma as a Public Health Problem in Amhara National Regional State, Ethiopia: Results of 152 Population-Based Surveys

**DOI:** 10.4269/ajtmh.19-0450

**Published:** 2019-09-23

**Authors:** Aisha E. P. Stewart, Mulat Zerihun, Demelash Gessese, Berhanu Melak, Eshetu Sata, Andrew W. Nute, Tigist Astale, Tekola Endeshaw, Tesfaye Teferi, Zerihun Tadesse, Elizabeth Kelly Callahan, Melsew Chanyalew, Birhan Gaudie, Paul M. Emerson, Jonathan D. King, Scott D. Nash

**Affiliations:** 1 The Carter Center, Atlanta, Georgia;; 2 The Carter Center, Bahir Dar, Ethiopia;; 3 The Carter Center, Addis Ababa, Ethiopia;; 4 International Trachoma Initiative, Addis Ababa, Ethiopia;; 5 Amhara National Regional Health Bureau, Bahir Dar, Ethiopia;; 6 Dr. Abdu Higher Eye Clinic, Bahir Dar, Ethiopia;; 7 International Trachoma Initiative, Atlanta, Georgia;; 8 World Health Organization, Geneva, Switzerland

## Abstract

At baseline in 2006, Amhara National Regional State, Ethiopia, was the most trachoma-endemic region in the country. Trachoma impact surveys (TIS) were conducted in all districts between 2010 and 2015, following 3–5 years of intervention with the WHO-recommended SAFE (surgery, antibiotics, facial cleanliness, and environmental improvement) strategy. A multistage cluster random sampling design was used to estimate the district-level prevalence of trachoma. In total, 1,887 clusters in 152 districts were surveyed, from which 208,265 individuals from 66,089 households were examined for clinical signs of trachoma. The regional prevalence of trachomatous inflammation-follicular (TF) and trachomatous inflammation-intense among children aged 1–9 years was 25.9% (95% CI: 24.9–26.9) and 5.5% (95% CI: 5.2–6.0), respectively. The prevalence of trachomatous scarring and trachomatous trichiasis among adults aged ≥ 15 years was 12.9% (95% CI: 12.2–13.6) and 3.9% (95% CI: 3.7–4.1), respectively. Among children aged 1–9 years, 76.5% (95% CI: 75.3–77.7) presented with a clean face; 66.2% (95% CI: 64.1–68.2) of households had access to water within 30 minutes round-trip, 48.1% (95% CI: 45.5–50.6) used an improved water source, and 46.2% (95% CI: 44.8–47.5) had evidence of a used latrine. Nine districts had a prevalence of TF below the elimination threshold of 5%. In hyperendemic areas, 3–5 years of implementation of SAFE is insufficient to achieve trachoma elimination as a public health problem; additional years of SAFE and several rounds of TIS will be required before trachoma is eliminated.

## INTRODUCTION

National trachoma programs conduct impact and surveillance surveys to assess the prevalence of clinical signs of trachoma and progress toward elimination as a public health problem. For trachoma, the targets for elimination as a public health problem include a prevalence of trachomatous inflammation-follicular (TF) among children aged 1–9 years of less than 5% at the health district level and a prevalence of trachomatous trichiasis (TT) unknown to the health system among the total population of less than one case per 1,000 at the health district level.^1^ Trachoma impact surveys (TIS) are presently conducted following 1–7 years of implementation of the WHO-endorsed surgery, antibiotics, facial cleanliness, and environmental improvement (SAFE) strategy^2^; however, previous guidance, followed until roughly 2017, called for TIS following 3–5 years of SAFE implementation.^3^

The National Survey on Blindness, Low Vision, and Trachoma, conducted in 2006, suggested Ethiopia to be the most trachoma-endemic country, among countries with available data. Within Ethiopia, the survey demonstrated that the Amhara Region harbored the highest regional prevalence of active trachoma (TF and/or trachomatous inflammation-intense [TI]) among children aged 1–9 years, 62.6%.^4^ Although trachoma interventions started in 2001 in Amhara, they were established in only four districts.^5^ Following the 2006 National Survey, further baseline data were collected during a 2007 zonal-level survey, which provided needed evidence to determine the zones (administrative unit below a region with about 2,000,000 population and made up of districts) that warranted SAFE intervention.

The 2007 baseline survey demonstrated that trachoma was endemic in all 10 zones in the region, each requiring full implementation of the SAFE strategy. Resultingly, SAFE interventions were gradually scaled up to all 152 districts between 2007 and 2010. Following 3–5 years of SAFE implementation, in accordance with global recommendations at the time, TIS were conducted in all districts between 2010 and 2015 to assess progress toward elimination.^6,7^ This article presents these aggregate TIS results, providing regional-, zonal-, and district-level summaries of progress toward trachoma elimination targets.

## MATERIALS AND METHODS

### Ethical considerations.

Survey methodology was approved by the Ethical Review Committee of the Amhara Public Health Research Institute, Ethiopia, and Emory University Institutional Review Board under protocol 079-2006. Permission to obtain verbal informed consent and assent was granted by the review boards because of the high rate of illiteracy among the study population. Verbal informed consent to conduct a household interview was obtained from heads of households or a representative 18 years or older. Verbal informed consent and assent were obtained from all individuals screened for trachoma and recorded on the household questionnaire. Permission to conduct the survey in the selected clusters was obtained from zonal and district health officials, and the cluster, or village, leader.

### Study site and time frame.

**Figure 1.**
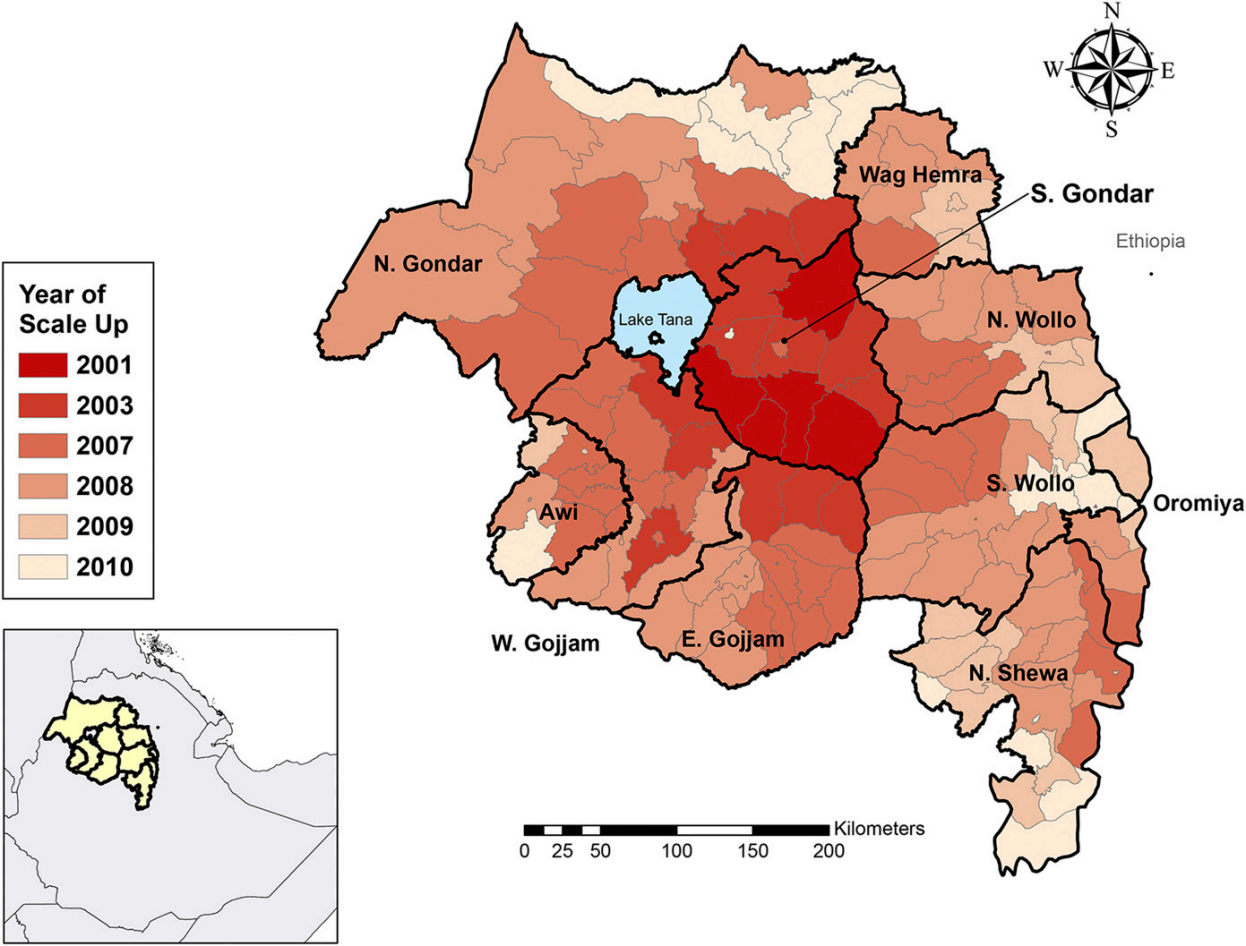
Scale-up of SAFE interventions by district, Amhara, Ethiopia, 2001–2010. This figure appears in color at www.ajtmh.org.

The trachoma program began in Amhara in 2001 in four districts and expanded to cover 19 districts in 2003 (Figure 1). Following the 2007 zonal-level baseline survey, SAFE interventions were gradually scaled up to all 152 districts between 2007 and 2010 (Table 1). This phased approach was used for logistical reasons, as providing trachoma services to an estimated 20 million residents^8^ in the region required substantial planning and resources. Areas with high zonal-level prevalence were prioritized for the early phase of scale-up, as it was assumed these areas would take longer to reach elimination thresholds. However, factors including geographic location, road access, and needs from the Regional Health Bureau also influenced the order in which SAFE interventions were rolled out to districts. Districts became eligible for TIS during different years between 2010 and 2015 because of the phased scale-up and varying baseline TF prevalence. In adherence with global guidelines, all TIS took place between 7 and 9 months after the last mass drug administration (MDA), with TIS occurring 8 months after MDA in 90 (59.2%) districts.

**Table 1:** Summary of regional SAFE activities implemented for trachoma control, Amhara, Ethiopia, 2007–2015^9–17^

Activity	Indicator	Regional output									Total
		2007	2008	2009	2010	2011	2012	2013	2014	2015	
Regional scale-up	Number of districts with SAFE	66	113	134	152	152	152	152	152	152	
Surgery: Awareness raising on the cause of TT and surgical approach to TT management; provision of TT surgical services through outreach camps and at health centers	Number of TT patients operated	45,271	31,561	35,681	33,021	39,076	66,766	44,867	40,450	71,460	408,153
Antibiotics: Annual dose of azithromycin or tetracycline eye ointment to all eligible community members	Number of doses of antibiotic distributed*	6,568,335	12,984,025	13,720,673	15,141,608	15,231,371	12,931,668	15,677,633	16,875,459	15,394,959	124,525,731
	% coverage	59.4%	116.0%	91.9%	93.7%	89.1%	95.4%	93.0%	100.1%	94.4%	
Facial cleanliness: Face washing and hygiene promotion in communities; school trachoma health curriculum	Number of villages with health education	1,447	2,898	3,432	3,428	3,427	3,449	3,459	3,459	3,459	
	Number of schools with health education	No school education.	6,181	6,922	6,935	7,884	8,374	8,374	8,374	2,553	
Environmental improvement: Promotion of household latrine construction and use	Number of household latrines constructed	466,359	373,677	544,205	590,119	284,423	520,885	282,078	104,777	305,511	3,472,034

TT = trachomatous trichiasis.

* Includes azithromycin and tetracycline eye ointment.

### Sampling methodology.

The TIS methodology used in Amhara between 2010 and 2015 evolved during that period to reflect best practices and innovation within the global trachoma program. As a result, three multistage, cluster random sampling methodologies were used. All surveys used the following: in the first stage, clusters, defined by a single *gott* (administrative unit comparable to a village), were selected from a geographically ordered list of all *gotts* using probability proportional to estimated population size. In the second stage, segments of households were selected once the field team arrived in the cluster. With the *gott* leader, or representative, the field team enumerated a list of development teams (official segments of about 40 households within *gotts*) and asked the *gott* leader to blindly pick the name of a segment from a hat.^18^ The *gott* leader or designated representative familiar with the village accompanied the field team to the randomly selected segment and during the fieldwork.

All households and household members within a selected segment were eligible to participate. If a household member older than 18 years was not present or if no one was at home when the field team arrived, the team made one attempt to return to the household to conduct an interview, and, if unsuccessful, the household was skipped without replacement. Children aged 1–9 years who were absent were added to a separate, electronic list in Swift Insights, the electronic data collection system, so that the survey team could return to the household once more for grading.

Differences in the sampling methodology occurred as follows: from 2010 to 2011, 25 districts were surveyed at the subdistrict level, as described previously.^19^ Between 2012 and 2015, 120 districts were surveyed, whereby the number of clusters selected in each district was proportional to the population of the district, and 40 households per development team were assumed. The remaining seven districts surveyed in 2015 used the same sample selection approach as the 2012–2015 TIS; however, the same number of clusters was chosen from each district, and, on average, 30 households per development team were assumed as evidence from previous surveys indicated these were rarely found to be as large as 40 households.

Among the 25 districts surveyed from 2010 to 2011, a sample size of 600 children aged 1–9 years was required to detect a prevalence of TF of 3% ± 2%, assuming 95% confidence, a design effect of two, five people per household with children aged 1–9 years representing 30% of the population, and allowing for an 8% nonresponse rate among children aged 1–9 years^20^; 10 clusters per subdistrict with 40 households each were randomly selected. Survey weights were used to aggregate data to the district and zonal levels.^19^

For 120 districts surveyed from 2012 to 2015, the sample size was calculated to detect a reduction in TF among children aged 1–9 years of at least 20% from baseline. Therefore, the greatest number of participants required was calculated using an assumed prevalence of TF among children aged 1–9 years of 50% and that children aged 1–9 years comprised 35% of the population. For an effect of at least 20% with a 95% level of significance, a design effect of five,^20^ 300 children aged 1–9 years per district were needed, requiring a sample of about 900 people of all ages per district.

For the final seven districts surveyed in 2015, a prevalence of 20% was assumed based on data from previous TIS in Amhara, where 137 of 145 districts maintained a TF prevalence greater than the elimination threshold of 5%. To detect a prevalence of 20% ± 10% from baseline with a 95% level of significance, a sample of approximately 215 children aged 1–9 years per district was estimated. This required a sample of about 615 people of all ages per district. This assumed a design effect of 3.04 and a 15% nonresponse rate, both of which had been calculated from previous TIS results from 145 districts in Amhara*.*

### Trachoma grading.

Before each round of TIS, trachoma graders were trained for 5–7 days, including those who had previously participated in TIS teams. Graders practiced grading at primary schools and at TT surgery camps to ensure exposure to all clinical signs of trachoma. Graders were required to pass both a written examination, identifying all trachoma clinical signs based on the WHO-simplified grading system^21^ (Table 2) using a set of 50 slides, and a field-reliability examination that included TF, TI, and trachomatous scarring (TS). Interobserver reliability for the field examination was assessed against concordant assessments from three master trainers. Graders achieving ≥ 84% agreement with TF and a κ ≥ 0.7 were eligible to participate in a survey team. Graders used 2.5 × binocular magnification loupes and appropriate light for screening.

**Table 2:** Definition of trachoma clinical signs

Trachoma clinical sign	Definition
Trachomatous inflammation-follicular	The presence of five or more follicles in the upper tarsal conjunctiva. Follicles are round swellings that are paler than the surrounding conjunctiva, appearing white, gray, or yellow. Follicles must be at least 0.5 mm in diameter.
Trachomatous inflammation-intense	Pronounced inflammatory thickening of the tarsal conjunctiva that obscures more than half of the normal deep tarsal vessels. The tarsal conjunctiva appears red, rough, and thickened. There are usually numerous follicles, which may be partially or totally covered by the thickened conjunctiva.
Trachomatous scarring	The presence of scarring in the tarsal conjunctiva. Scars are easily visible as white lines, bands, or sheets in the tarsal conjunctiva. They are glistening and fibrous in appearance. Scarring, especially diffuse fibrosis, may obscure the tarsal blood vessels.
Trachomatous trichiasis	At least one eyelash rubs on the eyeball. Evidence of recent removal of inturned eyelashes should also be graded as trichiasis.

Reproduced from Thylefors et al.^21^

### Questionnaires and trachoma examination.

Three questionnaires were used, including 1) *gott*-level interview with a community leader or representative, 2) household-level interview with a household representative older than 18 years, and 3) individual-level interview and trachoma screening. Each questionnaire was translated from English to Amharic then back-translated from Amharic to English. All surveys, except the first conducted in 2010 which was paper based, collected data electronically in Amharic using Swift Insights.^22^ Amharic questionnaires were used rather than English versions to ensure standardization among the way questions were asked, as the data recorders spoke limited English.

Data recorders received 5–7 days of training to learn to use the electronic data collection system and Android tablets, and to administer questionnaires following a standardized protocol. Data recorders pilot-tested questionnaires and use of the tablets in nonsurvey *gotts* to ensure quality and standardization of interviews and translation, as well as the functionality and usability of individual tablets and Swift Insights. Data recorders were required to pass an examination before the start of each survey to participate.

The *gott*-level interview collected information about the village, including the distance the survey team had to walk to reach the cluster from where the vehicle dropped them, and the presence of a paved road, electricity, mobile phone coverage, and a health facility in the community. The global positioning system coordinates of each cluster were taken using the tablet. The household interview was conducted at each selected and consenting household to gather information on socioeconomic status, water, and sanitation, as well as hygiene practices. Latrine presence and use were verified by observation by the data recorders.

All household members were enumerated and their name, age, and gender listed, regardless of whether they were present. Trained trachoma graders screened both eyes of all present household members for all clinical signs of trachoma.^21^ Trachoma graders assessed children aged 1–9 years for clean face, described as the absence of nasal and ocular discharge. Multiple teams worked in each district to reduce grader bias so that no one team surveyed all clusters in a district. Individuals presenting signs of active trachoma were offered treatment with 1% tetracycline eye ointment. Individuals with TT were referred to the nearest health center for surgical services. Questions to determine whether individuals with TT were known or unknown to the health system based on new recommendations from the WHO^23^ were included in the questionnaire for the final 37 districts surveyed. If an individual with unoperated TT was detected, then that person was asked if he/she had been offered surgery, with a response of “yes” indicating that the person was known to the health-care system.

Survey teams were supervised by dedicated TIS supervisors, all of whom worked for the regional trachoma program or the Carter Center. Each supervisor supported two to four teams per survey and accompanied the teams for the duration of the survey. Supervisors were trained before the start of each survey on the protocol. Supervisors’ primary role was to ensure survey teams conducted the survey according to the standardized protocol and to address issues as they arose.

### Data management and analysis.

Data were stored on the internal memory and on removable storage cards on the tablets and downloaded to password-protected computers by field supervisors every 2–5 days. Data from each enumerated individual were linked to the corresponding household-level data using a household serial number automatically generated by Swift Insights.

Statistical analysis was conducted using Stata version 14.0 (StataCorp, College Station, TX). Zonal and regional estimates were calculated, in some instances by aggregating data from multiple survey rounds. All estimates were calculated using the complex survey commands in Stata (svy) with weights created using the inverse of the sampling probability of both stages of sampling. Sampling weights allowed for district-level data to be aggregated to the zonal and regional levels. Robust CIs were estimated using Taylor-series linearization, which accounted for the clustering at household and cluster levels, and a finite population correction was specified for both stages of sampling.

## RESULTS

### Characteristics of study communities, households, and participants.

One thousand eight hundred eighty-seven clusters in 152 districts were surveyed. Among districts for which *gott*-level data were available (1,801), 30% of the sampled communities were located along a paved road, 11.5% had any community-level electricity supply, 66.2% had mobile phone coverage at the time the survey team visited, and 23.8% had a health facility. Survey teams recorded the time taken to walk from the vehicle to the cluster: 53.9% required less than a 1-hour walk, 25.8% required a 1- to 3-hour walk, and 20.4% required greater than a 3-hour walk.

Sixty-six thousand eighty-nine households were included in the survey. The prevalence of a functional household radio was 18.3% (95% CI: 17.4–19.2), a functioning TV 4.0% (95% CI: 3.4–4.7), working electricity in the home 11.4% (95% CI: 9.9–13.0), and a functioning mobile phone 20.2% (95% CI: 19.1–21.3). Corrugated iron or metal (54.5%) was the primary construction material making up the roof of the house, followed by thatch (38.9%).

Within the study clusters, 276,068 people were enumerated, and, among those, 208,265 people were examined for clinical signs of trachoma (Table 3), representing a 75.4% response rate. The average household size was 4.2 people. Children aged 1–9 years made up 29.0% of survey participants, 57.5% were adults aged ≥ 15 years, and 50.6% were female.

**Table 3:** Sample characteristics, Amhara, Ethiopia, 2010–2015

Zone	Survey date(s)	Number of districts	Number of clusters	Number of households	Number of people enumerated	People examined			
						Number of people	% Children aged 1–9 years	% Adults aged ≥ 15 years	% Females
Awi	June 2013	11	86	3,273	14,086	9,987	27.1	57.6	52.6
East Gojam	June 2013	18	176	6,820	27,961	20,158	25.1	60.0	53.1
North Gondar	May 2012, June 2013, October 2015	23	215	7,772	34,475	25,361	33.6	53.5	52.0
North Shoa	December 2012, January 2014, February 2015	24	196	7,521	30,081	21,292	23.6	61.6	50.5
North Wollo	December 2012, January 2014	12	147	5,756	21,998	16,683	24.8	61.0	51.9
Oromia	December 2012, January 2014	7	50	2,008	8,933	6,149	31.1	53.5	51.7
South Gondar	July 2011	12	354	12,012	50,884	38,652	36.4	54.0	47.6
South Wollo	December 2010, December 2012, January 2014, February 2015	22	461	13,415	55,267	46,112	24.9	59.7	49.1
Waghemra	December 2012, January 2014	7	55	2,095	8,794	6,747	30.7	55.4	51.2
West Gojam	May 2012, June 2013	16	147	5,417	23,589	17,124	31.6	56.0	52.2
Amhara Region		152	1,887	66,089	276,068	208,265	29.0	57.5	50.6

### Water, sanitation, and hygiene characteristics.

The round-trip time taken to collect water, including travel to the water source, queuing, collecting water, and returning home, was less than 30 minutes for 66.2% (95% CI: 64.1–68.2; Table 4) of households. This figure reflects a 10.7% decline from 74.1% at baseline in 2007.^7^ Households collecting water from an improved or a safe source increased to 48.1% (95% CI: 45.5–50.6) from 34.4% at baseline^7^; 50.2% (95% CI: 48.8–51.5) of households had a latrine present, a 106.6% increase from 24.3% at baseline^7^; and 46.2% (95% CI: 44.8–47.5) of households had evidence of latrine use. A hand-washing station was present in 13.9% (95% CI: 12.7–15.2) of households. Among children aged 1–9 years, 76.5% (95% CI: 75.3–77.7) had a clean face free of nasal and ocular discharge.

**Table 4:** Water, sanitation, and hygiene household characteristics, Amhara, Ethiopia, 2010–2015

Zone	Water access ≤ 30 minutes, % (95% CI)	Improved water source, % (95% CI)	Latrine present, % (95% CI)	Evidence of latrine use, % (95% CI)	Handwashing station, % (95% CI)	Clean face, % (95% CI)
Awi	76.0 (68.3–82.3)	47.2 (38.5–56.0)	76.4 (71.9–80.4)	71.0 (66.4–75.2)	24.0 (18.9–30.0)	72.9 (67.2–77.9)
East Gojam	73.4 (68.0–78.2)	43.6 (37.2–50.2)	53.3 (49.8–56.9)	50.0 (46.4–53.6)	12.0 (9.1–15.8)	76.6 (73.0–79.9)
North Gondar	64.2 (58.6–69.5)	40.7 (33.9–47.9)	28.7 (24.7–33.0)	24.9 (21.0–29.2)	8.9 (6.1–12.8)	78.3 (75.1–81.2)
North Shoa	56.0 (50.3–61.7)	54.0 (48.1–59.8)	52.7 (49.3–56.1)	48.1 (44.6–51.6)	20.4 (16.9–24.4)	78.0 (75.2–80.5)
North Wollo	48.5 (41.8–55.2)	61.3 (53.5–68.6)	60.9 (56.8–64.9)	56.1 (51.8–60.3)	18.8 (14.8–23.6)	78.1 (74.4–81.4)
Oromia	47.7 (37.5–58.1)	62.3 (49.3–73.8)	41.9 (34.5–49.8)	38.8 (31.8–46.2)	6.0 (3.1–11.4)	66.8 (61.6–71.6)
South Gondar	79.5 (75.2–83.2)	42.6 (37.0–48.3)	46.5 (42.9–50.1)	42.3 (38.6–46.0)	8.4 (6.3–11.2)	74.5 (70.6–78.1)
South Wollo	51.6 (43.2–60.0)	64.0 (54.7–72.3)	56.3 (53.5–59.1)	53.8 (51.0–56.7)	10.1 (8.5–12.1)	78.3 (76.3–80.1)
Waghemra	33.8 (24.1–45.2)	33.1 (22.3–46.0)	28.7 (22.9–35.3)	23.4 (17.8–30.1)	6.8 (3.4–13.1)	65.7 (58.3–72.5)
West Gojam	81.7 (76.5–85.9)	51.6 (42.8–60.3)	50.0 (45.1–54.8)	45.1 (40.3–50.0)	14.0 (9.8–19.5)	81.6 (78.5–84.3)
Amhara Region	66.2 (64.1–68.2)	48.1 (45.5–50.6)	50.2 (48.8–51.5)	46.2 (44.8–47.5)	13.9 (12.7–15.2)	76.5 (75.3–77.7)

### Trachoma prevalence.

The regional prevalence of TF among children aged 1–9 years was 25.9% (95% CI: 24.9–26.9; Table 5), a decrease of 33.8% from the National Survey in 2006^4^ and of 20.8% from the zonal baseline survey in 2007.^7^ The prevalence of TI among children aged 1–9 years saw the greatest decline among all trachoma clinical signs since baseline, decreasing 86.8% from 41.7% in 2006^4^ to 5.5% (95% CI: 5.2–6.0). The prevalence of active trachoma was 28.3% (95% CI: 27.3–29.3), a decrease of 54.8% from 62.6% in 2006.^4^ A lower zonal TF point estimate was observed at TIS than at baseline in seven of the 10 zones.

**Table 5:** Clinical signs of TF and TI among children aged 1–9 years at baseline (2007) and TIS (2010–2015), Amhara, Ethiopia

Zone	Baseline		TIS			
	No examined	TF	No examined	TF	TI	Active trachoma
		% (95% CI)		% (95% CI)	% (95% CI)	% (95% CI)
Awi	588	38.9 (22.7–57.9)	3,342	17.9 (14.9–21.3)	3.4 (2.5–4.6)	19.7 (16.6–23.1)
East Gojam	548	48.3 (44.4–52.2)	6,201	34.5 (31.7–37.4)	7.3 (6.0–8.8)	37.2 (34.3–40.1)
North Gondar	466	34.7 (24.4–46.8)	10,700	16.8 (14.3–19.7)	3.4 (2.5–4.5)	18.4 (15.8–21.4)
North Shoa	527	23.2 (14.1–35.9)	6,040	30.7 (27.6–33.9)	7.2 (5.8–8.8)	33.2 (30.1–36.5)
North Wollo	539	51.9 (35.4–68.0)	4,988	30.8 (26.8–35.1)	6.9 (5.5–8.4)	33.6 (29.5–37.9)
Oromia	663	28.7 (19.6–39.8)	2,290	35.1 (30.0–40.6)	3.7 (2.9–4.8)	36.4 (31.3–41.8)
South Gondar	589	28.9 (20.1–39.6)	17,561	25.9 (23.9–27.9)	7.0 (6.3–7.9)	29.7 (27.6–31.8)
South Wollo	484	12.6 (7.8–19.7)	13,035	24.3 (22.0–26.6)	4.1 (3.4–5.1)	25.8 (23.5–28.3)
Waghemra	581	60.1 (50.4–69.0)	2,440	54.7 (49.7–59.6)	13.7 (11.5–16.1)	59.3 (54.9–63.6)
West Gojam	500	33.1 (25.3–42.0)	6,884	18.2 (15.9–20.7)	3.4 (2.8–4.2)	20.6 (18.1–23.3)
Region	5,485	32.7 (29.2–36.5)	73,481	25.9 (24.9–26.9)	5.5 (5.2–6.0)	28.3 (27.3–29.3)

TF = trachomatous inflammation-follicular; TI = trachomatous inflammation-intense; TIS = trachoma impact surveys.

Trachomatous scarring prevalence among the total population was 7.7% (95% CI: 7.3–8.2) and 12.9% (95% CI: 12.2–13.6) among adults aged ≥ 15 years. Trachomatous trichiasis prevalence among the total population was 2.1% (95% CI: 2.0–2.2). Among adults aged ≥ 15 years, 3.9% (95% CI: 3.7–4.1) presented with TT, a decrease of 37.1% from 6.2% at baseline in 2007^7^ (Table 6). Zonal-level estimates of TT unknown to the health system could not be calculated, as these data were collected only for the final 37 districts surveyed, in accordance with recommendations from the WHO released in 2015,^23^ and estimates for all districts in any one zone were not available; however, the range by district was 0.0% to 5.0%. Eight of the 10 zonal TT point estimates were lower at TIS than at baseline.

**Table 6:** Clinical signs of TS and TT among adults aged ≥ 15 years at baseline (2007) and TIS (2010–2015), Amhara, Ethiopia

Zone	Baseline		TIS		
	No examined	TT	No examined	TS	TT
		% (95% CI)		% (95% CI)	% (95% CI)
Awi	893	5.4 (4.0–7.3)	5,242	11.5 (9.0–14.5)	3.5 (2.9–4.2)
East Gojam	881	7.1 (5.4–9.4)	11,165	11.3 (9.3–13.6)	5.9 (5.2–6.6)
North Gondar	730	4.3 (2.8–6.6)	11,979	13.3 (11.4–15.3)	3.2 (2.7–3.7)
North Shoa	943	9.0 (6.7–11.9)	12,625	10.7 (9.6–11.9)	4.2 (3.8–4.7)
North Wollo	971	9.4 (7.2–12.1)	9,566	11.9 (10.1–14.0)	4.0 (3.4–4.6)
Oromia	964	2.4 (1.4–4.1)	3,157	12.2 (10.3–14.3)	3.0 (2.5–3.7)
South Gondar	904	3.8 (2.5–5.7)	18,067	23.0 (20.8–25.4)	3.8 (3.4–4.3)
South Wollo	931	3.2 (2.2–4.6)	26,522	7.2 (6.3–8.3)	2.6 (2.3–3.0)
Waghemra	1,030	6.3 (3.9–9.9)	3,562	19.4 (16.3–23.0)	5.8 (4.8–7.1)
West Gojam	874	10.0 (6.3–15.6)	8,326	14.7 (12.3–17.4)	2.9 (2.4–3.6)
Region	9,121	6.2 (5.3–7.4)	110,211	12.9 (12.2–13.6)	3.9 (3.7–4.1)

TIS = trachoma impact surveys; TT = trachomatous trichiasis.

**Figure 2.**
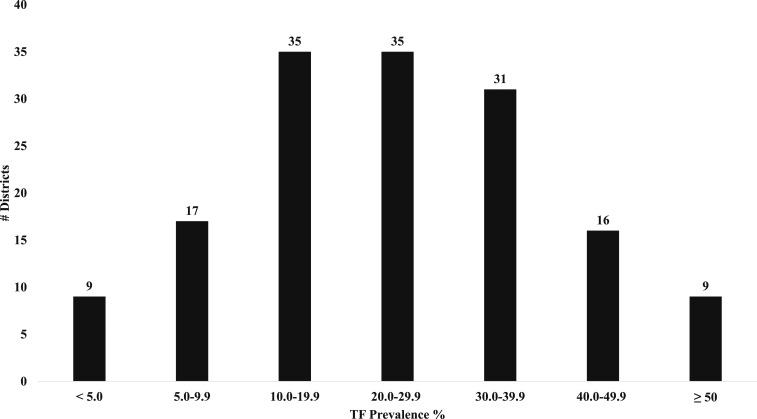
District counts of trachomatous inflammation-follicular prevalence among children aged 1–9 years, Amhara, Ethiopia, 2010–2015.

**Figure 3.**
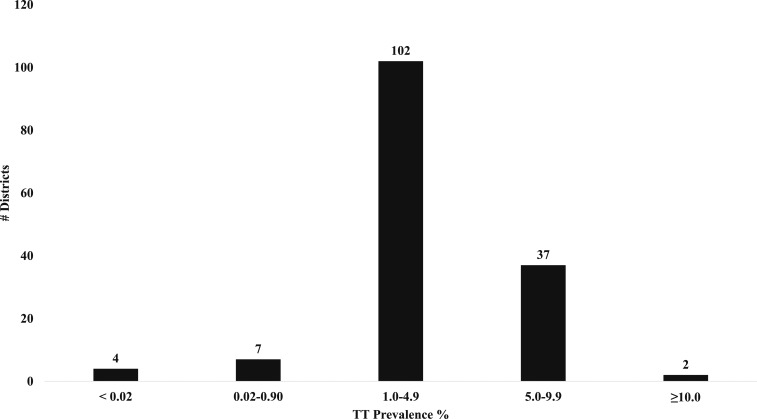
District counts of trachomatous trichiasis prevalence among adults aged ≥ 15 years, Amhara, Ethiopia, 2010–2015.

Substantial differences between zones were observed (Figures 2 and 3). North Gondar Zone presented the lowest prevalence of TF among children aged 1–9 years of 16.8% (95% CI: 14.3–19.7). Both North Gondar and Awi zones had the lowest prevalence of TI among children aged 1–9 years of 3.4%. Waghemra Zone had the highest prevalence of TF and TI among children aged 1–9 years of 54.7% (95% CI: 49.7–59.6) and 13.7% (95% CI: 11.5–16.1), respectively. South Wollo Zone evidenced the lowest prevalence of both TS and TT among adults aged ≥ 15 years of 7.2% (95% CI: 6.3–8.3) and 2.6% (95% CI: 2.3–3.0), respectively. East Gojam Zone had the highest prevalence of TT among adults aged ≥ 15 years of 5.9% (95% CI: 5.2–6.6), whereas South Gondar Zone harbored the highest prevalence of TS among adults aged ≥ 15 years of 23.0% (95% CI: 20.8–25.4).

Overall, nine districts achieved the TF target for elimination as a public health problem, whereas four districts achieved the target for TT (Figures 2 and 3, Supplemental Table 1). Among those districts, only two achieved both TF and TT targets. Seventy districts had an estimated prevalence of TF among children aged 1–9 years between 10% and 29.9%, whereas TF was hyperendemic (≥ 30%) in 56 districts. One hundred two districts presented a TT prevalence among adults aged ≥ 15 years between 1.0% and 4.9%, whereas 39 demonstrated a prevalence of at least 5.0%.

## DISCUSSION

Between 2010 and 2015, all 152 districts in the Amhara Region were surveyed to evaluate the SAFE strategy and assess progress toward elimination targets. Compared with baseline data, overall, these TIS demonstrated substantial uptake of SAFE interventions and successful reductions in the clinical signs of TF, TI, and TT.^4,7^ As a result of this work, Amhara became the first region in Ethiopia to complete TIS in every district.^24^

After the full recommended 3–5 years of SAFE was implemented throughout the region with high coverage, only nine districts met the elimination thresholds for TF, four met the TT targets, and two of these met the targets for both TF and TT. Substantial heterogeneity of trachoma prevalence among districts, even districts within the same zone, persists. Notably, 56 (37%) districts are hyperendemic, despite the SAFE activities. The results of these surveys make it clear that a combination or all S, A, F, and E activities continue to be warranted in 150 of 152 districts in Amhara, and that guidelines for SAFE interventions may need to be revisited for trachoma hyperendemic areas.

The TIS demonstrated an increase in the use of an improved water source from baseline, as well as a substantial increase in the presence of household latrines—both indications of some improvement in hygiene and sanitation developments that impact trachoma. Still, less than half of households reported collecting water from an improved water source and about 50% had an observed household latrine, highlighting the continued need for water, sanitation, and hygiene infrastructure. Continued health education, supported through delivery at schools and in the community, can also encourage the uptake of F and E components of the SAFE strategy.

Despite improvements in water and sanitation, the results from these surveys are consistent with those reported in other hyperendemic settings,^25,26^ in randomized trials,^27,28^ and in mathematical models.^29,30^ Unlike the success seen in hypoendemic and mesoendemic areas, evidence from hyperendemic settings highlight that 3–5 years of MDA may not be sufficient to reduce the prevalence of TF below elimination thresholds. Trachomatous inflammation-follicular has been shown to stabilize or return in high-burden areas despite annual or biannual MDA.^27,28,31^ Although evidence from clinical trials of biannual MDA did not show a statistically significant difference in efficacy to a single-round MDA, intensified MDA shows promise for accelerating the speed of decline of trachoma.^28,29^ Other treatment regimens, with either more intense, or better targeted MDA strategies should be tested.^28,32,33^

Although TF and TT are the indicators by which elimination as a public health problem is defined, evidence suggests that TI may be a better indicator of infection with *Chlamydia trachomatis*, the bacterium that causes trachoma. Trachomatous inflammation-follicular has also been shown to persist in areas despite low levels or no infection with *C. trachomatis*,^6,34,35^ purporting that TF may overestimate the presence of chlamydial infection in communities. Instead, studies have demonstrated a highly correlated relationship between TI and *C. trachomatis*.^6,34,36^ Future surveys should grade individuals for TI, as this clinical sign can be used as a proxy indicator for trachoma infection.

The TIS survey schedule recommended by the global program has evolved, shifting from 3–5 years following SAFE implementation, as established in 2010,^37,38^ to the current standard of 1–7 years, adopted in 2017. The exact number of years is dependent on the baseline prevalence of TF in the health district. The results from TIS in Amhara support the decision by the International Trachoma Initiative Trachoma Expert Committee to extend the number of years of SAFE implementation, including annual MDA in areas with baseline TF prevalence ≥ 50%, from 5 to 7 years,^39^ and align with previous evidence suggesting 7–10 years may be more appropriate.^26^ However, given the sheer scale of work required to complete TIS, enormous resources and time will be required for national programs to adhere to the presently recommended frequency of surveys. For example, almost one-half of the 1,887 clusters surveyed during the TIS described here required survey teams to walk more than 1 hour each way to reach the selected clusters.

Because multiple TIS and surveillance surveys are required in each health district, future survey designs must not only be scientifically sound but also be feasible to complete in a timely manner to also allow sufficient time during the year for SAFE strategy implementation. As the requirements for surveys increase as districts approach elimination targets, there is a risk that trachoma elimination programs become trachoma surveillance programs, which will do nothing to eliminate the disease.

These TIS provided robust prevalence estimates following full-scale implementation of the SAFE strategy. Compared with the 2007 zonal-level baseline, three zones evidenced a higher prevalence of TF among children aged 1–9 years, whereas one zone presented a higher prevalence of TT among adults aged ≥ 15 years. These apparent higher estimates of TF and TT may be due to methodological differences in the surveys, particularly because the magnitude of SAFE interventions delivered to these zones would make an increase in chlamydial infection unlikely. Interpreting different prevalence levels from baseline to TIS, both increases and decreases, will be a challenge that many trachoma programs will face globally as baseline surveys are often conducted using large enumeration units.^40,41^ Future district-level TIS in Amhara will allow for clearer assessments of progress toward trachoma elimination targets and uptake of SAFE interventions.

From 2010 to 2015, 152 districts, including 1,887 clusters throughout the Amhara Region, were surveyed to assess trachoma prevalence and the uptake of SAFE interventions. Two districts have met the trachoma elimination targets, whereas continued interventions are required in the remaining 150 districts. Trachoma impact surveys and then trachoma surveillance surveys have continued in eligible districts in Amhara. In areas with a high trachoma burden at baseline, like Amhara, trachoma interventions are likely to be required for a considerable period before the elimination targets are achieved.

## Supplemental table

Supplemental materials
