# Supplementary material for: Progress to Eliminate Trachoma as a Public Health Problem in Amhara National Regional State, Ethiopia: Results of 152 Population-Based Surveys
Source: Am J Trop Med Hyg. 2019 Sep 23;101(6):1286–95. doi: 10.4269/ajtmh.19-0450 (PMC6896880; doi:10.4269/ajtmh.19-0450)
Supplement: Supplementary file 1 [file tpmd190450.SD1.doc]

**Supplemental Table 1. District-level estimates of TF among children ages 1-9 years and TT among adults ≥ 15 years, Amhara, Ethiopia, 2010-2015.**

| **Zone** | **District** | **TF Prevalence among Children aged 1-9 years (95% CI)** | **TT Prevalence among Adults aged ≥ 15 years (95% CI)** |
| --- | --- | --- | --- |
| Awi | Ankesha | 23.4 (17.3-30.8) | 4.5 (3.0-6.8) |
| Awi | Banja | 30.6 (16.5-49.6) | 2.1 (0.7-6.2) |
| Awi | Chagni Town | 8.1 (1.8-30.1) | 1.7 (1.6-1.7) |
| Awi | Dangila Town | 14.3 (3.5-43.5) | 3.0 (1.2-7.0) |
| Awi | Dangila Zuria | 7.5 (4.3-12.7) | 2.4 (1.3-4.5) |
| Awi | Fagita Lekoma | 22.4 (15.1-31.9) | 2.3 (1.5-3.5) |
| Awi | Guagusa- Shikudad | 30.2 (17.8-46.3) | 3.9 (2.0-7.6) |
| Awi | Guangua | 15.4 (8.1-27.4) | 5.9 (3.6-9.5) |
| Awi | Injibara town | 1.6 (0.4-6.4) | 2.0 (1.4-2.8) |
| Awi | Jawi | 10.1 (5.0-18.9) | 2.4 (1.2-4.5) |
| Awi | Zigem | 16.4 (8.3-29.7) | 6.1 (3.8-9.8) |
| East Gojam | Aneded | 51.8 (37.3-66.1) | 5.1 (3.3-7.7) |
| East Gojam | Awabel | 36.1 (30.0-42.6) | 7.3 (5.0-10.5) |
| East Gojam | Baso Liben | 48.1 (32.9-63.5) | 5.5 (3.4-8.7) |
| East Gojam | Bibugn | 19.3 (9.5-35.0) | 2.2 (1.2-4.0) |
| East Gojam | Debay Tilatgin | 28.5 (18.6-41.0) | 3.7 (2.2-6.1) |
| East Gojam | Debre Eliase | 39.2 (28.5-51.0) | 6.8 (3.9-11.5) |
| East Gojam | Debre Markos Town | 22.9 (22.8-22.9) | 8.1 (8.0-8.1) |
| East Gojam | Dejene | 42.4 (25.1-61.7) | 6.4 (3.4-11.7) |
| East Gojam | Enarge | 41.8 (31.7-52.7) | 7.9 (5.0-12.2) |
| East Gojam | Enebssie Sar Midir | 33.8 (25.3-43.5) | 6.8 (4.2-10.7) |
| East Gojam | Enemay | 34.6 (26.4-43.9) | 6.5 (4.4-9.5) |
| East Gojam | Goncha | 25.7 (17.3-36.1) | 6.8 (4.6-10.1) |
| East Gojam | Gozamin | 35.8 (24.7-48.6) | 5.2 (3.7-7.4) |
| East Gojam | Huletu Eju Enessie | 33.8 (25.3-43.5) | 5.8 (3.8-8.6) |
| East Gojam | Machakel | 25.1 (14.3-40.2) | 4.1 (2.5-6.6) |
| East Gojam | Motta Town | 25.5 (11.3-47.9) | 3.2 (1.7-5.9) |
| East Gojam | Shebel | 39.8 (28.9-51.8) | 10.5 (8.1-13.5) |
| East Gojam | Sinan | 13.3 (5.7-28.6) | 1.9 (0.9-3.8) |
| North Gondar | Aderkay | 7.2 (3.4-14.6) | 5.7 (2.8-11.2) |
| North Gondar | Alefa | 19.2 (12.8-27.7) | 1.0 (0.3-3.0) |
| North Gondar | Beyeda | 26.2 (12.6-46.7) | 2.7 (0.9-7.7) |
| North Gondar | Chilga | 4.1 (2.1-7.9) | 1.4 (0.8-2.6) |
| North Gondar | Dabat | 42.8 (25.2-62.6) | 4.1 (1.9-8.7) |
| North Gondar | Debark Town | 11.3 (8.0-15.6) | 2.4 (1.2-4.9) |
| North Gondar | Debark Zuria | 23.4 (8.0-51.9) | 2.7 (1.7-4.3) |
| North Gondar | Dembia | 13.7 (7.1-24.7) | 2.5 (1.6-3.7) |
| North Gondar | East Belessa | 20.2 (10.7-35.0) | 6.8 (4.2-10.9) |
| North Gondar | Genda Wuha Town | 2.9 (0.4-18.8) | 0.9 (0.1-8.7) |
| North Gondar | Gondar Town | 25.7 (14.1-42.1) | 1.9 (0.5-7.6) |
| North Gondar | Gondar Zuria | 24.5 (15.8-36.0) | 2.9 (1.6-5.3) |
| North Gondar | Janamora | 36.0 (19.4-56.7) | 3.0 (1.2-7.2) |
| North Gondar | Lay Armachiho | 6.4 (2.5-15.4) | 3.3 (1.8-6.0) |
| North Gondar | Metema | 6.0 (2.9-12.2) | 2.4 (1.3-4.4) |
| North Gondar | Quara | 7.8 (3.2-18.0) | 2.4 (0.6-9.3) |
| North Gondar | Tach Armchiho | 2.5 (1.1-5.7) | 2.5 (1.3-4.8) |
| North Gondar | Takussa | 11.8 (7.2-18.6) | 1.3 (0.4-4.4) |
| North Gondar | Tegede | 3.6 (1.9-7.0) | 3.4 (1.9-6.1) |
| North Gondar | Telemt | 23.6 (12.8-39.5) | 6.6 (3.3-12.9) |
| North Gondar | West Armachiho | 8.4 (2.3-26.2) | 0.0 (0.0-0.0) |
| North Gondar | West Belessa | 41.4 (30.6-53.1) | 3.7 (1.9-6.9) |
| North Gondar | Wogera | 27.6 (20.2-36.4) | 4.2 (2.5-6.8) |
| North Shoa | Alem Ketema Town | 45.0 (30.1-60.9) | 8.1 (5.9-11.0) |
| North Shoa | Angolelanatera | 6.7 (2.9-14.7) | 1.3 (0.7-2.5) |
| North Shoa | Ankober | 43.6 (28.6-59.8) | 5.2 (3.4-8.1) |
| North Shoa | Antsokia Gemza | 28.6 (17.9-42.4) | 3.4 (2.1-5.3) |
| North Shoa | Asagirt | 12.8 (3.6-36.9) | 4.2 (1.7-10.0) |
| North Shoa | Basona Worana | 22.6 (13.7-34.9) | 2.2 (1.5-3.2) |
| North Shoa | Berehet | 4.8 (1.7-12.8) | 2.8 (0.9-8.1) |
| North Shoa | Debre Birhan Town | 5.1 (4.3-6.0) | 2.6 (1.1-6.1) |
| North Shoa | Efrata Gidim | 38.8 (30.6-47.6) | 7.2 (5.2-9.8) |
| North Shoa | Ensaro | 54.7 (39.7-69.0) | 10.4 (7.1-15.0) |
| North Shoa | Gishe | 31.3 (13.2-57.5) | 4.1 (1.9-8.4) |
| North Shoa | H/Mariam Kesem | 17.8 (7.9-35.5) | 2.3 (0.7-7.1) |
| North Shoa | Kewot | 18.5 (8.6-35.1) | 5.8 (4.0-8.3) |
| North Shoa | Menz-Gera | 12.0 (5.2-25.2) | 1.0 (0.4-2.3) |
| North Shoa | Menz-Keya | 44.9 (29.3-61.5) | 4.6 (2.4-8.5) |
| North Shoa | Menz-Lalo | 18.1 (9.0-33.0) | 1.7 (0.2-13.5) |
| North Shoa | Menz-Mama | 17.4 (6.1-40.4) | 1.1 (0.4-3.3) |
| North Shoa | Merhabete | 45.0 (30.1-60.9) | 8.1 (5.9-11.0) |
| North Shoa | Mida Woremo | 49.6 (37.1-62.1) | 5.9 (3.9-8.9) |
| North Shoa | Minjar Shenkora | 21.0 (15.7-27.6) | 3.5 (2.7-4.6) |
| North Shoa | Mojana Wodera | 33.5 (18.6-52.6) | 5.4 (2.5-11.0) |
| North Shoa | Moretna Jiru | 56.4 (42.1-69.6) | 3.4 (2.1-5.4) |
| North Shoa | Shoarobit Town | 27.3 (8.6-60.1) | 1.9 (0.4-8.0) |
| North Shoa | Siadebirnawayu | 27.5 (18.9-38.1) | 3.0 (1.7-5.2) |
| North Shoa | Tarmaber | 28.9 (13.0-52.6) | 4.5 (2.4-8.2) |
| North Wollo | Ayinabugina | 49.9 (31.1-68.6) | 8.7 (5.3-13.9) |
| North Wollo | Dawunt | 47.7 (30.5-65.4) | 5.9 (3.4-10.2) |
| North Wollo | Gidan | 34.4 (21.2-50.7) | 2.9 (1.9-4.5) |
| North Wollo | Gubalafto | 11.2 (6.5-18.5) | 2.4 (1.2-5.1) |
| North Wollo | Habiru | 14.1 (8.8-21.9) | 3.0 (2.0-4.5) |
| North Wollo | Kobo Town | 8.3 (1.8-30.9) | 2.7 (0.9-7.7) |
| North Wollo | Kobo Zuria | 36.0 (26.3-46.9) | 5.5 (4.1-7.3) |
| North Wollo | Lalibela Town | 31.1 (8.3-69.2) | 2.8 (1.2-6.3) |
| North Wollo | Lasta | 31.7 (17.7-49.9) | 5.3 (3.3-8.5) |
| North Wollo | Meket | 38.9 (29.5-49.3) | 4.1 (2.8-5.9) |
| North Wollo | Wadilla | 35.2 (19.4-55.0) | 2.4 (1.0-5.8) |
| North Wollo | Woldia | 8.5 (2.3-27.0) | 2.7 (1.2-5.9) |
| Oromia | Artuma Furse | 42.1 (30.0-55.1) | 2.7 (1.6-4.6) |
| Oromia | Bati Rural | 27.1 (17.6-39.2) | 4.3 (3.0-6.1) |
| Oromia | Bati Town | 12.9 (9.5-17.2) | 0.6 (0.1-3.7) |
| Oromia | Dawa Chafa | 41.3 (31.9-51.5) | 3.6 (2.6-5.0) |
| Oromia | Dawey Harawa | 37.6 (18.1-62.2) | 2.6 (0.8-8.4) |
| Oromia | Jille Tumuga | 33.0 (21.8-46.5) | 3.2 (1.8-5.6) |
| Oromia | Kemmissie | 29.9 (18.4-44.6) | 0.0 (0.0-0.0) |
| South Gondar | Debre Tabor town | 8.6 (3.8-18.3) | 2.2 (0.7-7.3) |
| South Gondar | Dera | 27.3 (21.2-34.4) | 3.0 (2.0-4.4) |
| South Gondar | East Estie | 18.9 (15.9-22.4) | 2.9 (1.9-4.5) |
| South Gondar | Ebinat | 44.9 (38.8-51.1) | 4.4 (3.1-6.2) |
| South Gondar | Farta | 17.8 (14.5-21.8) | 3.8 (2.9-5.1) |
| South Gondar | Fogera | 33.3 (25.7-41.8) | 3.4 (2.2-5.5) |
| South Gondar | Lay Gaynt | 18.8 (13.5-25.6) | 3.2 (2.2-4.8) |
| South Gondar | Libo Kemkem | 24.3 (17.9-32.2) | 3.1 (2.3-4.1) |
| South Gondar | Simada | 23.9 (19.9-28.4) | 7.2 (5.2-9.8) |
| South Gondar | Tach Gaynt | 33.8 (23.0-46.6) | 4.0 (2.4-6.6) |
| South Gondar | West Estie | 30.6 (23.7-38.5) | 4.2 (2.8-6.3) |
| South Gondar | Woreta town | 17.2 (10.2-27.5) | 2.4 (0.9-6.3) |
| South Wollo | Albuko | 1.0 (0.3-2.7) | 0.0 (0.0-0.0) |
| South Wollo | Ambassel | 14.7 (6.8-28.7) | 5.4 (2.8-10.3) |
| South Wollo | Argoba | 37.2 (20.6-57.4) | 2.6 (1.4-4.8) |
| South Wollo | Borena/Debresinna | 15.1 (12.2-18.5) | 2.2 (1.5-3.2) |
| South Wollo | Delanta | 25.9 (14.5-41.9) | 3.2 (1.8-5.7) |
| South Wollo | Dessie Ketema | 9.4 (2.9-26.1) | 2.5 (1.1-5.7) |
| South Wollo | Dessie Zuria | 15.4 (9.0-25.1) | 2.1 (1.3-3.3) |
| South Wollo | Jamma | 21.8 (13.2-34.0) | 2.9 (1.7-4.8) |
| South Wollo | Kalala | 28.9 (22.0-37.0) | 4.0 (3.0-5.2) |
| South Wollo | Kalu | 24.0 (18.6-30.3) | 3.0 (1.9-4.8) |
| South Wollo | Kombolcha | 10.7 (4.9-21.8) | 4.4 (1.4-13.3) |
| South Wollo | Kutaber | 21.7 (15.3-29.7) | 1.4 (0.7-2.9) |
| South Wollo | Legambo | 20.5 (15.1-27.3) | 0.5 (0.2-1.1) |
| South Wollo | Legehida | 67.0 (54.0-77.8) | 2.3 (1.4-3.7) |
| South Wollo | Mehal Sayient | 2.2 (1.0-4.6) | 2.0 (1.0-3.7) |
| South Wollo | Mekidella | 9.5 (5.6-15.5) | 0.7 (0.3-1.8) |
| South Wollo | Sayient | 30.7 (20.2-43.7) | 3.7 (2.5-5.5) |
| South Wollo | Tehuledere | 5.7 (3.0-10.5) | 3.0 (2.0-4.6) |
| South Wollo | Tenta | 8.5 (5.5-12.9) | 1.8 (1.0-3.3) |
| South Wollo | Wogide | 30.9 (23.7-39.2) | 5.6 (4.4-7.1) |
| South Wollo | Worebabo | 36.7 (22.5-53.7) | 3.3 (2.2-4.9) |
| South Wollo | Woreilu | 46.5 (40.1-53.1) | 1.7 (1.0-2.9) |
| Waghemra | Abergelle | 73.9 (49.0-89.3) | 3.5 (1.5-7.8) |
| Waghemra | Dahanna | 62.0 (54.4-69.1) | 6.9 (5.3-8.9) |
| Waghemra | Gazegibilla | 59.0 (48.1-68.9) | 3.9 (1.9-8.0) |
| Waghemra | Sahalla Seyemt | 15.4 (10.7-21.7) | 5.1 (2.7-9.4) |
| Waghemra | Sekota Ketema | 65.3 (47.0-80.0) | 9.0 (2.4-28.5) |
| Waghemra | Sekota Zuria | 55.6 (44.9-65.7) | 6.5 (4.6-9.1) |
| Waghemra | Ziqualla | 33.9 (22.6-47.4) | 4.5 (2.4-8.3) |
| West Gojam | Bahir Dar City | 5.4 (2.8-10.0) | 3.6 (1.5-8.2) |
| West Gojam | Bahir Dar Zuria | 14.4 (7.2-26.7) | 1.7 (0.6-4.8) |
| West Gojam | Bure Zuria | 31.1 (16.7-50.3) | 7.4 (4.4-12.3) |
| West Gojam | Dega Damot | 18.6 (11.6-28.6) | 1.5 (0.8-2.8) |
| West Gojam | Dembecha | 27.0 (14.9-43.9) | 3.4 (1.8-6.2) |
| West Gojam | Finot Selam Town | 0.0 (0.0-0.0) | 0.0 (0.0-0.0) |
| West Gojam | Gonje Kollela | 23.2 (16.0-32.2) | 6.5 (3.9-10.8) |
| West Gojam | Jabi Tinan | 19.0 (11.9-29.1) | 3.9 (2.2-6.8) |
| West Gojam | Mecha | 15.7 (11.9-20.4) | 3.7 (2.4-5.8) |
| West Gojam | North Achefer | 12.5 (9.7-16.1) | 2.4 (1.4-3.9) |
| West Gojam | Quarit | 21.0 (12.9-32.2) | 2.6 (1.4-4.8) |
| West Gojam | Sekella | 20.4 (14.3-28.1) | 3.1 (1.6-5.8) |
| West Gojam | South Achefer | 14.3 (6.3-29.2) | 3.2 (1.7-5.8) |
| West Gojam | Wonberma | 30.9 (22.0-41.5) | 3.6 (1.1-10.7) |
| West Gojam | Yilmana Denssa | 19.2 (14.3-25.2) | 0.4 (0.1-1.5) |
